# Adhesion G protein-coupled receptor latrophilin 1 (ADGRL1): a novel regulator of glucose and energy homeostasis

**DOI:** 10.1038/s41392-024-01925-x

**Published:** 2024-07-30

**Authors:** Kavaljit H. Chhabra

**Affiliations:** Department of Pharmacology and Nutritional Sciences, Barnstable Brown Diabetes and Obesity Research Center, University of Kentucky, Lexington, KY USA

**Keywords:** Physiology, Endocrine system and metabolic diseases

Recently, I read with interest the research article entitled ‘*Dysfunction of the adhesion G protein-coupled receptor latrophilin 1 (ADGRL1/LPHN1) increases the risk of obesity*’^1^ published in *Signal Transduction and Targeted Therapy*. This report confirms and extends our findings that ADGRL1 (aka latrophilin 1) regulates energy balance and glucose homeostasis.^2^ Using affinity chromatography coupled with proteomics and electrophysiology, we showed that ADGRL1 is likely a glucose receptor in the hypothalamus. Our findings demonstrated that lack of ADGRL1 in the hypothalamus or whole-body causes obesity and features of type 2 diabetes in mice. It is refreshing to see Dietzsch et al.^1^ reached the same conclusion.

ADGRL1 is widely studied as a receptor for alpha-latrotoxin^3,4^ and in neuroscience research in the context of synaptic activity. We are beginning to determine the contribution of the central and peripheral ADGRL1 to glucose sensing, development of obesity and type 2 diabetes.^1,2^ Interestingly, clinical features in a cohort of humans with pathogenic ADGRL1 variants show that four out of nine individuals with the variants were overweight.^5^ Similarly, Dietzsch et al.^1^ also identified variants of the human LPHN1 that contribute to the obesity phenotype.

In the interest of the readers and audience in obesity and diabetes research, I would like to take this opportunity to highlight some of the differences in the models used by Dietzsch et al.^1^ and Chhabra et al.^2^:

1. Dietzsch et al. used constitutive *Adgrl1* knockout mice throughout their study. In contrast, Chhabra et al. used conditional (ventromedial hypothalamus-specific) Adgrl1^VMH^ knockout mice for the majority of their study. In this model, Adgrl1 was knocked out only in the VMH using the Cre-Lox recombination approach and other tissues had normal Adgrl1 expression. This may explain why Dietzsch et al. did not observe the sex differences in the obesity and associated phenotype in their global *Adgrl1* knockout mouse model.
2. For producing global *Adgrl1* deficiency, Chhabra et al. used the transcriptional block (loxTB) strategy, in which global *Adgrl1* gene remains unexpressed (similar to a knockout model) but allows for a potential expression of *Adgrl1* in a Cre-dependent manner in desired tissues. We do observe obesity phenotype in both male and female loxTB-*Adgrl1* mice (unpublished data from our laboratory), which is comparable to that reported by Dietzsch et al.
3. Dietzsch et al. used a transient transfection approach in cell lines to confirm the glucose-mediated changes in cAMP levels published by Chhabra et al. who used a stable cell line. The stable cell lines offer more consistent results because the gene of interest (*Adgrl1* in this case) is integrated into the host genome and they are gold standards in drug discovery and development. In addition, changes in cAMP levels are not a readout for glucose sensing, but more of a downstream second messenger mechanisms triggered by glucose–ADGRL1 binding. Chhabra et al. demonstrated the glucose sensing property of hypothalamic ADGRL1 using brain slice electrophysiology and through observing glucose-induced changes in food intake in *Adgrl1*^VMH^ knockout mice. Despite this evidence, considering the manuscript published by Dietzsch et al., further investigation into glucose-ADGRL1 downstream signaling pathway is warranted.

**Fig. 1:**
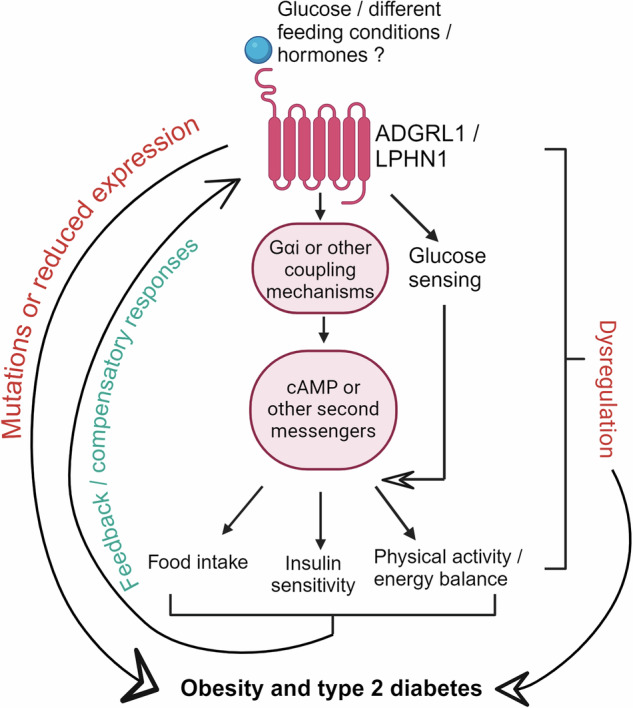
Role of adhesion G protein-coupled receptor latrophilin 1 (ADGRL1/latrophilin 1/LPHN1) in regulating glucose and energy homeostasis through multiple potential pathways/mechanisms. Created with BioRender.com

Further studies will determine whether ADGRL1 is regulated by changes in food intake, physical activity, and/or by fluctuations in blood glucose levels (Fig. 1) to establish its role in common (multifactorial/polygenic) obesity versus rare (monogenic) forms of obesity. Different models of obesity and diabetes mellitus including multiple paradigms of fasting and feeding conditions will help address these research questions. Similarly, given its high expression in the hypothalamus and its glucose sensing property,^2^ investigating the role of hypothalamic ADGRL1 in detecting and counteracting hypoglycemia is warranted. This will determine the contribution of the receptor to hypoglycemia unawareness and/or impaired counterregulatory responses observed in humans with type 1 diabetes. It is likely ADGRL1 interacts with leptin and estrogen receptors in the VMH to differentially influence energy and glucose homeostasis in male and female animals. Finally, it is intriguing that the expression of genes encoding traditional orexigenic or anorexigenic peptides such as *Pomc*, *Cck*, *Npy*, and *Agrp* were not changed in *Adgrl1* knockout mice,^1^ but circulating levels of ghrelin and GLP1 were reduced in the knockout mice. It remains unclear to what extent ADGRL1 regulates energy and glucose homeostasis through its yet unknown independent signaling pathways and/or via a crosstalk with the known regulators of energy balance such as leptin, ghrelin, and GLP1.

In summary, both the recent reports,^1,2^ which are the topic of discussion of this correspondence, concluded ADGRL1 is a novel regulator of food intake, body weight, and glucose homeostasis (Fig. 1). Therefore, ADGRL1 appears to be a therapeutic target for treatment of obesity and type 2 diabetes.
